# Evaluating ChatGPT and Google Gemini Performance and Implications in Turkish Dental Education

**DOI:** 10.7759/cureus.77292

**Published:** 2025-01-11

**Authors:** Ipek Kinikoglu

**Affiliations:** 1 Pedodontics, Istanbul Turkuaz Dental Clinic, Istanbul, TUR

**Keywords:** artificial intelligence in dentistry, chatgpt, chatgpt conventional teaching dental students, dentistry, gemini advanced

## Abstract

Artificial intelligence (AI) has emerged as a transformative tool in education, particularly in specialized fields such as dentistry. This study evaluated the performance of four advanced AI models - ChatGPT-4o (San Francisco, CA: OpenAI), ChatGPT-o1, Gemini 1.5 Pro (Mountain View, CA: Google LLC), and Gemini 2.0 Advanced, in the Turkish Dental Specialty Examination (DUS) for 2020 and 2021. A total of 240 questions, comprising 120 questions per year from basic and clinical sciences, were analyzed. AI models were assessed based on their accuracy in providing correct answers compared to the official answer keys.

For the 2020 DUS, ChatGPT-o1 and Gemini 2.0 Advanced achieved the highest accuracy rates of 93.70% and 96.80%, respectively, with net scores of 112.50 and 115 out of 120 questions. ChatGPT-4o and Gemini 1.5 Pro followed with accuracy rates of 83.33% and 85.40%. For the 2021 DUS, ChatGPT-o1 again demonstrated the highest accuracy at 97.88% (115.50 net score), closely followed by Gemini 2.0 Advanced at 96.82% (114.25 net score). Overall, ChatGPT-4o and Gemini 1.5 Pro scored lower for 2021, achieving accuracy rates of 88.35% and 93.64%, respectively.

Combining results from both years (238 total questions), ChatGPT-o1 and Gemini 2.0 Advanced achieved accuracy rates of 97.46% (230 correct answers, 95% CI: 94.62%, 100.00%) and 97.90% (231 correct answers, 95% CI: 94.62%, 100.00%), respectively, significantly outperforming ChatGPT-4o (88.66%, 211 correct answers, 95% CI: 85.43%, 91.89%) and Gemini 1.5 Pro (91.60%, 218 correct answers, 95% CI: 87.75%, 95.45%). Statistical analysis revealed significant differences among the models (p = 0.0002). Pairwise comparisons demonstrated that ChatGPT-4o underperformed significantly compared to ChatGPT-o1 (p = 0.0016) and Gemini 2.0 Advanced (p = 0.0007) after Bonferroni correction. The consistently high accuracy rates and narrow confidence intervals for the top-performing models underscore their superior reliability and performance in answering the DUS questions.

Generative AI modules such as ChatGPT-01 and Gemini 2.0 have the potential to enhance dental board exam preparation through question evaluation. While the AI modules appear to outperform humans on DUS questions, the study raises a concern about the ethical uses of AI and the true justification and value of DUS examinations as dental competency examinations. A higher level of knowledge evaluation should be considered. This research contributes to the growing body of literature on AI applications in specialized knowledge domains and provides a foundation for further exploration of its integration into dental education.

## Introduction

Dental education requires postgraduate specialty training to ensure that dentists possess comprehensive knowledge and skills in oral and dental health. In Turkey, the Dental Specialty Examination (DUS) is a centralized examination for the placement of dentists in specialty programs. This examination assesses the clinical and theoretical knowledge of dental students and determines their eligibility for specialty training [1]. DUS covers a broad range of topics in basic and clinical dental sciences, requiring candidates to undergo a comprehensive preparation process. In recent years, the use of artificial intelligence (AI) technologies in education and health has increased. The ability of AI to analyze large datasets, recognize patterns, and answer complex questions offers potential benefits in the exam preparation and evaluation processes [2].

Advances in AI and machine learning have led to significant developments in areas such as natural language processing (NLP) and deep learning [3]. These advances have enabled the development of AI models capable of understanding and interpreting complex texts. These models have shown promising results in various fields, including answering questions in the medical field, diagnosis, and treatment planning [4]. In particular, large language models (LLMs) and transformer-based architectures have demonstrated impressive performance in medical examinations such as the United States Medical Licensing Examination (USMLE) and the national board exam [5,6]. Specifically, ChatGPT 4.0 (San Francisco, CA: OpenAI) demonstrated high accuracy in cardiovascular clinical cases from the USMLE Step 2CK and Step 3, achieving a 90.3% accuracy rate, suggesting its effectiveness as a study resource [7]. Additionally, GPT-4 has been noted for its proficiency in handling USMLE-standard questions, with accuracy rates between 80% and 90% [8]. This has laid the groundwork for investigating the capacity of AI to answer questions in dentistry.

Recent studies have demonstrated that AI models such as ChatGPT 3.5 and 4 can achieve high accuracy rates on US dental exams like the Integrated National Board Dental Examination (INBDE), Dental Admission Test (DAT), and Advanced Dental Admission Test (ADAT), particularly in knowledge-based and comprehension questions [9]. However, these AI models face challenges, especially with questions involving complex figures or images, which are common in prosthodontic and restorative dentistry sections [10,11]. Despite these challenges, AI's potential to enhance dental education and examination processes is significant, suggesting a need for continued research and adaptation in educational practices to leverage AI's capabilities while addressing its limitations.

This study evaluated the performance of AI in answering the DUS questions from 2020 to 2021. The questions from the DUS exams in these years were presented to the AI models, and the models' correct answer rates and performances were analyzed. This research aimed to determine the potential use of AI in dental education and evaluation processes and to contribute to the development of AI-supported educational tools. The integration of AI into dental education has the potential to offer personalized learning experiences, provide objective assessments, and support the professional development of dentists [12]. Additionally, AI-powered question-answering systems can provide dental students with additional resources when preparing for exams and make their learning processes more effective [13].

However, the use of AI in education and evaluation raises both ethical and practical concerns [14]. The reliability, transparency, and impartiality of AI models are critical for their widespread adoption [15]. Furthermore, large and high-quality datasets are required for the training and development of AI models. These datasets must be updated regularly to reflect current knowledge and best practices in the field of dentistry [16]. This study aimed to assess the contribution of artificial intelligence models, particularly generative AI such as ChatGPT and Gemini (Mountain View, CA: Google LLC), in dental exams and to evaluate their potential role in enhancing the education and preparation of dental students for competency-based examinations.

## Materials and methods

This study evaluated the performance of four different artificial intelligence models in answering questions from the Turkish Dental Specialty Examination (DUS) administered in 2020 and 2021 [17,18].

Data acquisition

The questions, corresponding correct answers, and associated images from the 2020 and 2021 DUS exams were obtained from the official website of the Turkish Measuring, Selection, and Placement Center (ÖSYM), the organization responsible for administering the DUS [17,18]. The questions were extracted in their original format, including the color images presented in the official exam PDFs. The questions and images were presented to the AI models exactly as presented by ÖSYM. A total of 240 questions, 120 questions from each year were used in this study. The exams were designed such that four incorrect answers would cancel out one correct answer, adding an additional layer of complexity to the assessment. The questions were categorized into basic and clinical science.

AI models

Four AI models were selected for the study. Model 1 was OpenAI's ChatGPT (GPT-4o), accessed through the OpenAI website and used with the default settings [19]. Model 2 was OpenAI's ChatGPT (o1), also accessed via the OpenAI website, with default settings applied [20]. Model 3 was Google Gemini 1.5 Pro, accessed through Google’s AI Gemini website and used at the default settings without any fine-tuning. Model 4 was Google Gemini Advanced (2.0 experimental version), an advanced experimental version of the Gemini model, accessed via Google AI and used in its default state to explore its capabilities [21].

Question input and response collection

The 2020 and 2021 DUS questions were individually inputted into each AI model. Each question was presented as a separate query that included an associated image from the original PDF. For consistency, each question was posed in the following format: "[Question Text] A) [Option A] B) [Option B] C) [Option C] D) [Option D] E) [Option E].” In addition, each prompt included the instruction: "Please analyze the provided image along with the question to provide your best answer." The images were uploaded to each question using the native image upload functionalities of the respective AI platforms. The answers provided by the AI models were verbatim.

Performance evaluation

The responses generated by each AI model were compared with the official answer keys provided by ÖSYM. The primary performance metric was accuracy, calculated as the percentage of questions answered correctly by each model. Accuracy was calculated for each year's exam and for each dental discipline separately. Additionally, an error analysis was performed to identify the types of questions that the models struggled with, including those with image components.

To gain a deeper understanding of the models’ performance, an error analysis was conducted by subgrouping the questions based on the number of incorrect answers. The subgroups included the following: one mistake - questions that were incorrectly answered by only one model; two mistakes - questions that were incorrectly answered by two models; three mistakes - questions that were incorrectly answered by three models; and four mistakes - questions that were incorrectly answered by all four models.

This subgrouping allowed us to identify specific patterns of difficulty across questions and to investigate whether certain types of questions posed consistent challenges for the models. Special attention was given to questions with image components, as these were excluded from the analysis due to the inherent limitations of the models in processing visual data.

Descriptive statistics were used to summarize the accuracy of each AI model. The chi-square test was used to compare the performance of the models between the two years (2020 and 2021) and between different dental disciplines. To account for multiple comparisons in the post hoc pairwise analysis, the Bonferroni correction was applied. The adjusted significance level was set at 0.0083 (0.05, divided by six comparisons). A p-value of less than 0.05 (prior to Bonferroni correction) was considered statistically significant. Python version 3.13.1. was used for the statistical analysis.

Ethical considerations

This study utilized publicly available data from the ÖSYM website and did not involve human subjects. Therefore, ethical approval was not required for this study.

## Results

The performance of the four artificial intelligence models, ChatGPT-4o, ChatGPT-o1, Gemini 1.5 Pro, and Gemini 2.0 Advanced, was assessed using their scores from the 2020 and 2021 Turkish Dental Specialty Examinations. 

Performance on the 2020 DUS Examination

In the 2020 DUS, ChatGPT-o1 and Gemini 2.0 Experimental Advanced achieved the highest net scores, with 112.50 and 115 net scores, with six and four wrong answers out of 120 questions, respectively. ChatGPT-4o and Gemini 1.5, followed by net scores of 100 and 102.50, with 16 and 14 mistakes, respectively (Table 1).

**Table 1:** Performance of AI models on the 2020 DUS Examination. DUS: Turkish Dental Specialty Examination; AI: artificial intelligence

Model	Basic science questions (total: 40)	Clinical science questions (total: 80)	Net score	Overall accuracy (%, total: 120)
ChatGPT-4o	39	65	100	83.33%
Gemini 1.5 Pro	38	68	102.5	85.4%
ChatGPT-o1	40	74	112.5	93.70%
Gemini 2.0 Advanced	40	76	115	96.8%

Statistical analysis demonstrated a significant overall difference in the performance among the models (p = 0.010). However, pairwise comparisons did not reveal any statistically significant differences after applying Bonferroni correction (0.0083), suggesting that performance differences are not robust under stricter statistical thresholds.

Performance on the 2021 DUS Examination

The results for the 2021 DUS ChatGPT-o1 achieved the highest overall accuracy, with a 115.50 net score out of 118 questions (two wrong answers), followed by Gemini 2.0 Advanced with 114.25 (three wrong answers), Gemini 1.5 Pro with 110.50 (six wrong answers), and ChatGPT-4o with 104.25 (11 wrong answers) (Table 2).

**Table 2:** Performance of AI models on the 2021 DUS Examination. *Two questions from the 2021 DUS clinical sciences were officially canceled and excluded from the total count. DUS: Turkish Dental Specialty Examination; AI: artificial intelligence

Model	Basic science questions (total: 40)	Clinical science questions (total: 78)*	Net score	Overall accuracy (%, total: 118)
ChatGPT-4o	39	68	104.25	88.35%
Gemini 1.5 Pro	40	72	110.50	93.64%
ChatGPT-o1	40	76	115.50	97.88%
Gemini 2.0 Advanced	40	75	114.25	96.82%

Statistical analysis revealed an overall difference in the performance among the models (p = 0.025). However, none of the pairwise comparisons were statistically significant after applying the Bonferroni correction.

Combined performance across 2020 and 2021 DUS Examinations

**Figure 1:**
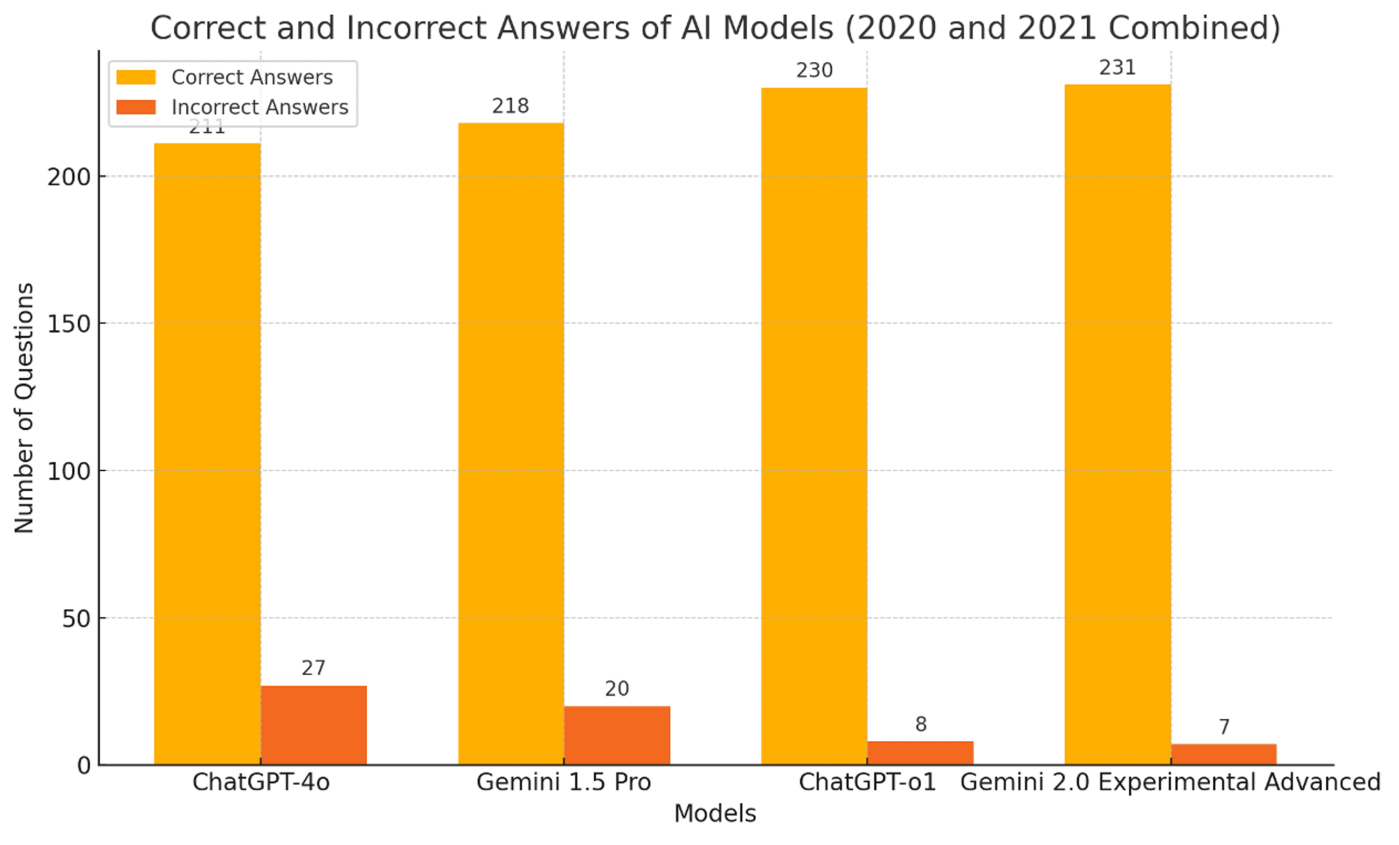
Correct and incorrect answers of AI models (2020 and 2021 combined). AI: artificial intelligence

The performance of the four AI models was assessed across a total of 238 questions from the 2020 and 2021 DUS examinations. ChatGPT-o1 and Gemini 2.0 Advanced achieved the highest number of correct answers, with 230 and 231, respectively, corresponding to only eight and seven incorrect answers. In comparison, Gemini 1.5 Pro answered 218 questions correctly with 20 incorrect responses, while ChatGPT-4o demonstrated the lowest performance with 211 correct answers and 27 incorrect responses (Figure 1).

The combined performance of the models across the 2020 and 2021 examinations revealed significant differences in the accuracy (p = 0.0002). Pairwise comparisons demonstrated that ChatGPT-4o performed significantly worse than both ChatGPT-o1 (p = 0.0016) and Gemini 2.0 Advanced (p = 0.0007), after Bonferroni correction (Table 3). However, no significant differences were observed between ChatGPT-4o and Gemini 1.5 Pro (p = 0.3566) or among the remaining pairwise comparisons, including Gemini 1.5 Pro vs. ChatGPT-o1 (p = 0.0321) and Gemini 1.5 Pro vs. Gemini 2.0 Advanced (p = 0.0174).

**Table 3:** Pairwise comparisons of AI model performance on DUS questions. DUS: Turkish Dental Specialty Examination; AI: artificial intelligence

Comparison	Chi-square (χ²)	Adjusted p-value (Bonferroni < 0.0083)
ChatGPT-4o vs. Gemini 1.5 Pro	0.8499	0.3566
ChatGPT-4o vs. ChatGPT-o1	9.9918	0.0016
ChatGPT-4o vs. Gemini 2.0 Experimental Advanced	11.4344	0.0007
Gemini 1.5 Pro vs. ChatGPT-o1	4.5915	0.0321
Gemini 1.5 Pro vs. Gemini 2.0 Advanced	5.654	0.0174
ChatGPT-o1 vs. Gemini 2.0 Advanced	0.0	1.0

Mistake analysis

The mistake analysis for the AI models on the 2020 and 2021 DUS questions provides valuable insights into their performance and areas for improvement. A significant number of errors in both years occurred with multi-part questions that required distinguishing between nuanced statements rather than providing direct answers. For instance, in 2021, one question, which involved evaluating statements about cervicofacial actinomycosis, challenged all four models. This question required careful interpretation of clinical details and the logical relationships between the statements. Similarly, in 2020, a question about the suitability of specific dental conditions for single-session root canal treatment was incorrectly answered by three models (ChatGPT-4o, Gemini 1.5 Pro, and Gemini 2.0 Advanced). In addition to text-based challenges, image-based questions also contributed significantly to errors. In 2020, a single figure-based question was correctly answered by all models, demonstrating their proficiency with simpler visuals. However, in 2021, the six-figure-based questions revealed notable difficulties as follows: ChatGPT-4o made errors on four, Gemini 1.5 Pro on two, and ChatGPT-o1 and Gemini 2.0 Advanced each made one mistake, though on different visuals. This indicates that while AI models perform well with straightforward visuals, they struggle with complex image-based scenarios requiring the integration of visual and textual information.

## Discussion

This study evaluated the performance of four advanced AI models - ChatGPT-4o, ChatGPT-o1, Gemini 1.5 Pro, and Gemini 2.0 Experimental Advanced - on Turkish Dental Specialty Examinations from 2020 to 2021. The results indicated that while all models demonstrated a high level of proficiency in answering dental specialty questions, there were notable differences in their overall accuracy and consistency. These findings have profound implications for the use of AI as a transformative tool in dental education.

A key finding was the superior performance of ChatGPT-o1 and Gemini 2.0 Advanced, which not only consistently outperformed the other two models but also surpassed the highest scores achieved by human candidates in both 2020 (108) [22] and 2021 (109.25) [23]. In the 2020 DUS examination, ChatGPT-o1 and Gemini 2.0 Experimental Advanced, achieved net scores of 112.50 and 115, respectively, exceeding the top human score. This landmark achievement demonstrates that these AI models possess capabilities that exceed human performance levels in this specific context. This exceptional performance underscores the potential of AI models to serve as powerful educational tools capable of providing dental students with a level of support and guidance that may surpass traditional learning methods.

These results align with the reported proficiency of GPT-4 in both the US Integrated National Board Dental Examination (INBDE) and UK Overseas Registration Examination (ORE), where it achieved pass rates of 80.7% and 62.7%, respectively, surpassing GPT-3.5 and human graduate benchmarks​ [10]. Notably, GPT-4's consistent performance in "Biostatistics, Experimental Design, and Data Analysis" (93.33%) and "Therapy" (69.12%) highlights its versatility and relevance across foundational and clinical domains.

In the findings of the present study, ChatGPT-o1 and Gemini 2.0 Experimental Advanced not only outperformed human participants but also set benchmarks for AI proficiency in high-stakes, specialized examinations. Similarly, ChatGPT-4.0's significant leap over ChatGPT-3.5, correctly answering 80.7% of US and 62.7% of UK exam questions, showcases the trajectory of AI evolution. The higher accuracy of ChatGPT-4.0 in addressing complex questions, reflected in its ability to correctly answer 327 more questions than its predecessor, resonates with the trend of improved question-specific accuracy seen in Gemini 2.0 Experimental Advanced's 96.8% overall accuracy in this study [24].

The superior performance of these models aligns with the growing body of literature that highlights the potential of AI in medical education and assessment. For instance, recent studies have demonstrated the ability of LLMs to achieve passing scores on medical licensing examinations, such as the United States Medical Licensing Examination (USMLE) [25,26]. Kung et al. found that ChatGPT achieved or exceeded the passing threshold for all three USMLE examinations without specific training [25]. Similarly, Gilson et al. reported that several LLMs performed comparably to medical students in clinical reasoning tasks [26]. However, these studies have mainly focused on the medical domain, and this study contributes to the limited amount of research in dentistry. The success of ChatGPT-o1 and Gemini 2.0 Experimental Advanced on the DUS further supports the notion that AI can effectively navigate specialized knowledge domains.

The success of ChatGPT-o1 and Gemini 2.0 Experimental Advanced can be attributed to the sophisticated algorithms and deep learning techniques employed. These models likely leverage architectures such as those used in other successful dental AI applications, like diagnostic charting in panoramic radiography, which has achieved high sensitivity and precision in detecting dental conditions [27,28]. These applications often utilize convolutional neural networks (CNNs) and other advanced machine learning techniques that excel in tasks such as detecting dental caries and other oral health issues [29]. The proficiency of these models in processing complex dental knowledge suggests that they can be adapted to create interactive and personalized learning experiences for students.

The performance of AI models, particularly ChatGPT-o1 and Gemini 2.0 Advanced, offers a compelling glimpse into the transformative potential of artificial intelligence in dental education. These models can revolutionize the learning experience by addressing various educational needs and challenges, thus making education more personalized, accessible, and efficient. They can tailor content to individual needs, offer targeted support for areas of weakness, such as customized practices for endodontic procedures, and enable adaptive assessments that adjust difficulty based on performance for accurate evaluations.

Their 24/7 availability allows students to learn at their convenience, whereas real-time feedback helps identify and correct mistakes instantly. Future developments may include simulated clinical scenarios to provide a safe environment for students to practice diagnostic and treatment planning skills. Moreover, these models can democratize education by offering high-quality resources to underserved regions, thereby breaking the barriers to dental training. By integrating AI into dental education, these advancements promise to enhance accessibility, efficiency, and learning outcomes for students worldwide.

This study had several limitations. First, the sample size, consisting of two years of DUS examinations, was relatively small. Future studies should incorporate larger datasets. Second, the study focused solely on multiple-choice questions. Incorporating case-based scenarios or open-ended questions can provide a more comprehensive evaluation. The generalizability of these findings may be limited by the focus on a single exam in one country. Finally, the difficulty of the DUS exam may not be representative of other board exams, which could explain the strong performance of the models.

Future research should focus on developing and refining AI-powered educational tools that leverage the capabilities demonstrated in this study. Investigating the explainability of AI responses is crucial for building trust and ensuring the responsible use of these technologies in educational settings [4]. Furthermore, studies examining the impact of AI-assisted learning on student outcomes, long-term knowledge retention, and clinical performance are essential to validate the effectiveness of these tools.

## Conclusions

In conclusion, this study highlights the potential of advanced AI models, particularly ChatGPT-o1 and Gemini 2.0 Advanced, to perform at a level comparable to or surpassing top human candidates on dental specialty examinations. These findings suggest that AI could transform dental education by offering personalized, adaptive, and efficient learning tools. However, the models also demonstrated notable weaknesses, particularly in handling nuanced multi-part questions, logical reasoning, and integrating complex visual and textual information. These limitations emphasize the need for further refinement of algorithms and more diverse training datasets to enhance their reliability and applicability.

Despite their promise, these models should be viewed as complementary tools rather than replacements for human expertise. The study's reliance on a specific dataset limited to DUS questions raises questions about generalizability to broader dental practice scenarios. Ethical concerns, such as transparency in AI decision-making and the risk of over-reliance on technology, must also be addressed. Future research should expand the evaluation scope, improve reasoning and interpretive capabilities, and explore their impact on learning outcomes. By balancing these advancements with caution, AI can responsibly enhance dental education and practice without compromising the critical role of human judgment.
